# Assessment of the Acute Toxicity and Anxiolytic‐Like Effect of *α‐*Humulene in Adult Zebrafish (*Danio rerio*)

**DOI:** 10.1002/cbdv.71534

**Published:** 2026-07-26

**Authors:** Alex de Souza Borges, Italo Moura Barbosa, Jéssica Bezerra Maciel, Aluísio Marques da Fonseca, Jesyka Macedo Guedes, Jane Eire Silva Alencar de Menezes, Gyllyandeson de Araujo Delmondes, Marta Regina Kerntopf, Hélcio Silva dos Santos, Alexandre Magno Rodrigues Teixeira

**Affiliations:** ^1^ Department of Chemical‐Biology, Post‐Graduate Program in Chemical‐Biology Regional University of Cariri, Pimenta Campus Crato Ceará Brazil; ^2^ Laboratory of Chemical‐Pharmacology and Environmental Bioassays, Post‐Graduate Program in Natural Sciences State University of Ceará Fortaleza Ceará Brazil; ^3^ Graduate Program in Sociobiodiversity and Sustainable Technologies University of International Integration of Afro‐Brazilian Lusophony Redenção Ceará Brazil; ^4^ Nursing Collegiate, Petrolina Campus Federal University of São Francisco Vale Petrolina Pernambuco Brazil

**Keywords:** alpha‐humulene, anxiolytic‐like effect, natural product, sesquiterpene, zebrafish

## Abstract

The sesquiterpene α‐humulene (alpha‐humulene, humulene, or HUM) is recognized for its anti‐inflammatory actions. However, its pharmacological potential in the central nervous system (CNS) remains largely unexplored. In this study, we evaluated the acute toxicity and behavioral responses of adult zebrafish (*Danio rerio*) to intraperitoneal (IP) injection of HUM. The behavioral parameters examined were number of line crossings (open‐field test) and time spent in the light zone of the tank (light/dark test). Then were performed experiments with standard antagonists to investigate the possible mechanism of action. Humulene exhibited low acute toxicity (LD_50_ > 40 mg kg^−1^, IP) and produced a maximal anxiolytic‐like effect at an intermediate dose of 20 mg kg^−1^ IP. The pharmacological dissection revealed that this effect was independent of the benzodiazepine (B_ZD_) allosteric binding site on the gamma‐aminobutyric acid (GABA) type A receptor (GABA_A_R), but it was completely reversed by granisetron (G_RAN_), a serotonin (5‐HT) type 3 receptor (5‐HT_3_R) antagonist. Further in silico analysis revealed a low‐affinity binding and an allosteric interaction of α‐humulene, respectively, with GABA_A_R and 5‐HT_3_R channels. Together, these results suggest a noncanonical mechanism involving both GABAergic and serotonergic systems.

## Graphical abstract

The monocyclic sesquiterpene α‐humulene elicited a significant anxiolytic‐like behavior in adult zebrafish and no observable acute toxicity. Pharmacological dissection suggested that this effect is independent of the benzodiazepine (B_ZD_) binding site on the gamma‐aminobutyric acid (GABA) type A receptor (GABA_A_R), but it depends on the serotonin type 3 channel receptor (5‐HT_3_R) activation. Further molecular docking procedure showed nonpolar interactions of this compound with 5‐HT_3_R.

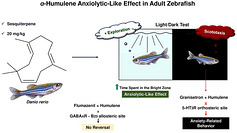

## 1 Introduction

Natural products of plant origin constitute a primary source for the discovery and development of new drugs, perpetuating a millennial therapeutic tradition in the treatment of various pathologies. In this context, essential oils stand out for their diverse chemical composition and broad pharmacological potential. Among their constituents, terpenes and terpenoids are the largest class of bioactive metabolites with ecological importance and therapeutic potential. Going beyond empirical use, research seeks to elucidate the mechanism of action of these compounds, demonstrating their specific interactions in living systems and consolidating their therapeutic applications [1, 2, 3, 4, 5, 6]. Structurally, these components consist of repeating isoprene units, which are five‐carbon hydrocarbons [1, 3, 5], and have been shown to induce positive behavioral responses in preclinical models of human neuropsychiatric disorders [7, 8, 9, 10, 11, 12, 13, 14]. They are classified by the number of isoprene units they contain (e.g., monoterpenes have two units). About one‐quarter of the terpene fractions in these oils are monoterpenes and sesquiterpenes. Notably, sesquiterpenes, which are formed from three linked isoprene units, are the most diverse subclass of terpenes and can have acyclic, monocyclic, bicyclic, or tricyclic structures [1, 3, 5, 15].

A candidate from this subclass is alpha‐humulene (α‐humulene). Plants containing this compound are used to treat insomnia, depression, nervousness, delirium, anxiety, and digestive disorders, and some of these are also important for the pharmaceutical and beverage industries. HUM is generally present alongside its more prominent isomer, *β*‐caryophyllene (BCP), and isocaryophyllene. BCP stands out in the literature across various preclinical models, which include studies using zebrafish, and exhibits anxiolytic, antidepressant, and anticonvulsant activities [7, 8, 9, 10, 11, 16, 17, 18, 19, 20, 21, 22, 23, 24, 25, 26, 27, 28, 29, 30, 31].

The literature on humulene indicates its involvement in proliferative cellular pathways, which are linked to potential treatments for inflammatory and neoplastic diseases. In practical applications, this terpene serves as a marker in the essential oil of the medicinal plant “erva‐baleeira” (*Varronia curassavica* Jacq. Cordiaceae), the active ingredient in Acheflan, a Brazilian topical anti‐inflammatory phytopharmaceutical [16, 17, 18, 19, 20, 21, 22, 23, 24, 25, 26, 27, 28, 29, 30, 31]. This highlights HUM's direct role in the therapeutic efficacy of this medicinal product.

Even in this context, there is a research gap regarding the action of humulene in behavioral disease models, which prompted the conduct of this work. In vitro and rodent in vivo assays are the primary sources demonstrating the pharmacological potential of this terpene, but few evaluate its action on CNS targets and behavioral parameters [32, 33, 34, 35, 36, 37]. Moreover, data on its toxicological profile and safety are scarce [16, 18].

Translational rodent models are useful tools for studying diseases; however, they are limited by high cost and low throughput. For example, the housing and chronic treatment of these animals with drugs is expensive and time‐consuming. Thus, the selection of complementary models in research on the phenotype of neuropsychiatric disorders, such as anxiety, and their conserved mechanisms is encouraged [38].

Over the last three decades, research using the *Danio rerio* (*D. rerio*) model has deepened the understanding of the neurobiology of vertebrate behavior and human neurological diseases. This species exhibits genetic homology with mammals. Genome sequencing has shown that 70% of human genes have at least one ortholog in zebrafish, and 84% of genes associated with human diseases are conserved in zebrafish [39]. This vertebrate is currently the second‐most‐used laboratory animal (after the mouse), with a well‐defined behavioral phenotype and established paradigms for its study. This species has low maintenance costs, rapid development, high fecundity, ease of drug administration, and similarity to mammals in the morphology and functions of various tissues and organs, including the brain and its main circuits: neurotransmitter systems and evolutionarily conserved stress‐related neuroendocrine mechanisms [40, 41]. From this perspective, this study aimed to evaluate the acute toxicity and characterize the anxiolytic potential of α‐humulene using adult zebrafish as a translational model for behavioral and pharmacological screening.

## 2 Results and Discussion

### 2.1 Acute Toxicity Evaluation

This protocol evaluated the acute toxic effect of humulene at three doses in adult zebrafish. The doses chosen were 4, 20, and 40 mg kg^−1^ (HUM‐4, HUM‐20, and HUM‐40, respectively). Following the HUM intraperitoneal (IP) injections, no death occurred in the 96 hour period, and the fish did not present any apparent anatomical or behavioral altered feature (*p* > 0.05). The median (Mdn) lethal dose was estimated to be greater than 40 mg kg^−1^ (LD_50_> 40 mg kg^−1^).

Information on α‐humulene toxicity is limited [16, 18]. According to Chaves et al., it has rapid topical and oral absorption but low oral bioavailability, which can be justified by its extensive first‐pass metabolism [34, 42, 43, 44, 45, 46]. In vivo and in vitro pharmacokinetic evidence indicate high initial hepatic concentrations, rapid clearance, and intense enzymatic modulation (significant inhibition or induction of phase I enzymes and strong induction of phase II enzymes) in human, rat, and mouse liver and microsomal models [43, 44, 45]. Two hours after IP injection, HUM concentration in mouse plasma was almost undetectable. This reflects its lipophilic nature, which suggests rapid diffusion across biological membranes, as well as rapid metabolism. Its elimination half‐life was 118.5 min for the oral route and 55 min for the intravenous route [42]. This rapid elimination can be interpreted as a positive sign, as it may reduce the risks of pronounced systemic toxicity. Also, it is important to note that the HUM's action on hepatic enzymes is inferred as potentially chemopreventive.

Related to behavioral analysis, Schwarz et al. reported that oral gavage (OG) of HUM at 200 or 500 mg kg^−1^ had no impact on locomotor activity nor nociception in mice, causing small but significant hypothermia [34]. Intratracheal administration in rats caused no mortality but resulted in pulmonary hemorrhage, though the damage extent was not visually pronounced [46]. In an antitumor treatment protocol, this component caused weight loss in mice [47], whereas in a model of airway inflammation, treated animals showed weight gain similar to the control group (untreated), supporting a low side‐effect profile [48].

In vitro evidence suggests that humulene has specific cellular targets for its toxic activity, with effects that are concentration‐dependent across different cell lines [19, 47, 49, 50]. It demonstrated selective cytotoxicity for neoplastic cells [16, 18, 19, 20, 21], but in normal human neurons, it induced an increase in reactive oxygen species (ROS) and a reduction in glutathione, with loss of cell viability after 24 h of incubation [33]; in human mast cells, its toxic action was considered minimal [51]. In mouse microglia, it reduced viability by 30%–50% after three hours of incubation [34], but in rat astrocytes, it decreased ROS production and increased antioxidant defenses [37].

Furthermore, this sesquiterpene has insecticidal, larvicidal, and molluscicidal action and suggests selectivity for these invertebrate targets, as it did not affect the survival or the swimming activity of the species *Gambusia affinis* (mosquitofish) at concentrations effective against the target mosquito larvae [52, 53, 54, 55, 56].

In summary, the doses of α‐humulene used in *D. rerio* were found to be safe based on their observed acute toxicity. These data establish, for the first time, a safety window for acute doses up to 40 mg kg^−1^ (IP) in this in vivo model, providing a parameter for future investigations. Despite ambiguous reports in isolated cell cultures, the compound had no observable toxicity in adult zebrafish.

### 2.2 Locomotor Activity Assessment

This protocol evaluated the effect of the selected humulene doses, the same as those used in the acute toxicity assay, on the locomotor activity of adult zebrafish in the open field test. The parameter observed was the number of line crossings. In addition to the group for each dose tested, two control groups were also analyzed for comparison. Diazepam (D_ZP_), standard anxiolytic B_ZD_ drug) was used as the positive control and the vehicle solution of 3% dimethyl sulfoxide (DMSO_3%_) as the negative control. After treatments, each animal was observed in an open‐field apparatus for 5 min (0–300 s).

**FIGURE 1:**
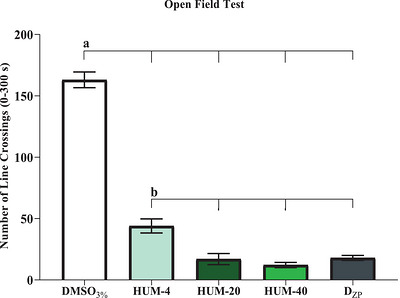
Effect of α‐humulene on the Locomotor Activity of Adult Zebrafish in the open field test. Values are expressed as mean plus‐minus (±) standard error of the mean (SEM); six fishes per group (*n* = 6). Statistical analysis was performed using one‐way ANOVA followed by Tukey's post–hoc test. Letters indicate the order of statistically significant comparisons among groups. (a): DMSO_3%_ versus HUM‐4 (*p* < 0.001; *g* = 9.87); DMSO_3%_ versus HUM‐20 (*p* < 0.001; *g* = 12.11); DMSO_3%_ versus HUM‐40 (*p* < 0.001; *g* = 12.52); and DMSO_3%_ versus D_ZP_ (*p* < 0.001; *g* = 12.03). (b): HUM‐4 versus HUM‐20 (*p* < 0.01; *g* = 2.24); HUM‐4 versus HUM‐40 (*p* < 0.001; *g* = 2.64); HUM‐4 versus D_ZP_ (*p* < 0.01; *g* = 2.15).

Figure 1 shows the results for the effect of humulene on the locomotor activity of adult zebrafish in the open field test. The one‐way ANOVA indicated a statistically significant difference among groups, *F*(4,25) = 198.3; *p* < 0.001; *ω* ^2^ = 0.963 (95% CI: 0.930 to 0.977). Tukey's post–hoc comparisons showed that the DMSO_3%_ group had a higher number of crossings than the groups treated with humulene doses or D_ZP_, **a**: DMSO_3%_ versus HUM‐4 (Δ*M* = 119, 95% CI: 100.14 to 137.85; *p* < 0.001; *g* = 9.87); DMSO_3%_ versus HUM‐20 (Δ*M* = 146, 95% CI: 127.14 to 164.85; *p* < 0.001; *g* = 12.11); DMSO_3%_ versus HUM‐40 (Δ*M* = 150.83, 95% CI: 131.98 to 169.69; *p* < 0.001; *g* = 12.52); and DMSO_3%_ versus D_ZP_ (Δ*M* = 145, 95% CI: 126.14 to 163.85; *p* < 0.001; *g* = 12.03).

Comparison among the doses demonstrated greater reduction in line crossings as the dose increased. HUM‐4 caused a less pronounced reduction, while HUM‐40 produced the greatest reduction in crossings, b: HUM‐4 versus HUM‐20 (Δ*M* = 27, 95% CI: 8.14 to 45.85; *p* < 0.01; *g* = 2.24); HUM‐4 versus HUM‐40 (Δ*M* = 31.83, 95% CI: 12.98 to 50.69; *p* < 0.001; *g* = 2.64); HUM‐20 and HUM‐40 did not differ significantly from each other. The positive control D_ZP_ was also more effective in reducing line crossings than humulene lowest dose, b: HUM‐4 versus D_ZP_ (Δ*M* = 26, 95% CI: 7.14 to 44.85; *p* < 0.01; *g* = 2.15). HUM‐20 or HUM‐40 did not differ significantly from D_ZP_.

The open field test allows evaluation of innate behavioral tendencies in zebrafish, such as neophobia and thigmotaxis (aversion to novel environments and the center of the apparatus), as well as the animal's general locomotor activity [57, 58, 59, 60, 61, 62, 63, 64]. This reflects the conserved evolution between this vertebrate and mammals, including deficits in the control of these behaviors, and validates it as a translational model for screening psychoactive drugs [65, 66, 67, 68, 69, 70, 71]. The number of line crossings is an indicator of exploratory behavior that can be related to anxiety. A fish that crosses the line repeatedly may be hyperactive due to handling or a new environment, or may be high‐exploring due to low anxiety. On the other hand, immobility can be interpreted as a reflection of freezing associated with anxiety or as sedation resulting from a treatment, leading to ambiguous interpretations [57, 58, 59, 60, 61, 62, 63].

All three doses of humulene reduced the number of line crossings made by zebrafish in a monotonic dose‐response fashion. This significant reduction in locomotor activity aligns with findings for its biogenic relative BCP, which has a sedative effect in *D. rerio* [8, 9]. In rodents, LaVigne et al. reported that mice treated with HUM (IP) alone exhibited moderate cataleptic behavior—mostly mediated by adenosine type two A receptor (A2A) and partially by endocannabinoid type one receptor (CB1)—however, the hypolocomotor measurements had no statistical significance [35]. Additionally, Schwarz et al. did not observe altered motor response in mice to humulene treatment (OG) [34], and Seekins et al. results for the acute antinociceptive effect of HUM (IP) do not suggest a sedation and/or motor suppression confound in mice [72]. The dose‐dependent reduction in the number of crossings indicates that α‐humulene modulates the exploratory activity of the animals, which may reflect both an anxiolytic effect and adverse motor sedation. Given this, the light/dark test was employed as a complementary tool to allow specific dissociation between these behavioral components, as widely validated in the literature for this species [57, 58, 59, 60, 61, 62, 63].

### 2.3 Anxiolytic‐Like Effect Assessment

This protocol evaluated the anxiolytic response of adult zebrafish to humulene at chosen doses in the light/dark test (0–300 s). The control groups used for comparisons were the same as previously mentioned. The parameter observed was the time spent in the tank's light zone. The translational effect of a substance may be determined by changes in zebrafish scototaxis (preference for the dark zone of the tank), as the aversion to the bright environment reflects the approach‐avoidance conflict characteristic of anxiety models. Thus, the effects of anxiolytic drugs are observed by an increase in the time spent in the bright zone of the tank [57, 58, 59, 60, 61, 62, 63, 64, 65, 66, 67, 68, 69, 70, 71].

**FIGURE 2:**
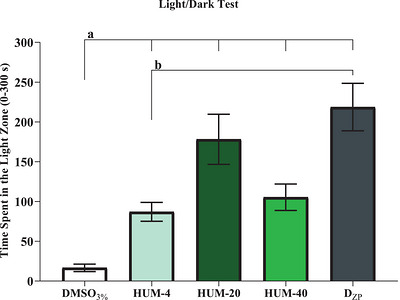
Anxiolytic‐like effect of α‐humulene in adult zebrafish in the light/dark test. Values are expressed as mean ± SEM; *n* = 6. Statistical analysis was performed using Welch's ANOVA followed by Games–Howell's post–hoc test. Letters indicate the order of statistically significant comparisons among groups. (a) DMSO_3%_ versus HUM‐4 (*p* < 0.01; *e* = 5); DMSO_3%_ versus HUM‐20 (*p* < 0.05; *e* = 5.42); DMSO_3%_ versus HUM‐40 (*p* < 0.05; *e* = 5.27); and DMSO_3%_ versus D_ZP_ (*p* < 0.01; *e* = 4.62). (b) HUM‐4 versus D_ZP_ (*p* < 0.05; *e* = 38.98).

Figure 2 shows the investigation results on the anxiolytic‐like effect of α‐humulene in adult zebrafish using the light/dark test. Welch's ANOVA indicated a statistically significant difference among groups, *F*(4,11) = 23.42; *p* < 0.001; *ω* ^2^ = 0.625 (95% CI: 0.319 to 0.760). The Games–Howell post–hoc test showed that treatments increased time spent in the light zone compared to the negative control group. The comparisons were as follows, **a**: DMSO_3%_ versus HUM‐4 (Δ*M* = −70.33, 95% CI: −116.97 to −23.69; *p* < 0.01; *e* = 5); DMSO_3%_ versus HUM‐20 (Δ*M* = −161.50, 95% CI: −287 to −35.96; *p* < 0.05; *e* = 5.42); DMSO_3%_ versus HUM‐40 (Δ*M* = −88.67, 95% CI: −154.13 to −23.20; *p* < 0.05; *e* = 5.27); and DMSO_3%_ versus D_ZP_ (Δ*M* = −202, 95% CI: −320.89 to −83.10; *p* < 0.01; *e* = 4.62).

Humulene reduced the anxiety parameter in zebrafish similarly to the positive control D_ZP_, with a statistically significant difference observed only with the lowest dose HUM‐4, in which the standard anxiolytic drug promoted a greater increase in the time spent in the light zone of the tank, **b**: HUM‐4 versus D_ZP_ (Δ*M* = −131.67, 95% CI: −248.86 to −14.47; *p* < 0.05; *e* = 38.98). No significant differences occurred between HUM‐20 and HUM‐40, nor did HUM‐20 and HUM‐40 differ significantly from D_ZP_. The effect size for HUM‐20 (*e* = 5.42) was greater than that of D_ZP_ (*e* = 4.62), which highlights the significance of this result. Although the difference between 20 and 40 mg kg^−1^ was not statistically significant, the graph suggests a non‐monotonic dose‐response, with the anxiolytic effect reaching a plateau or even decreasing at the highest dose. This may result from reduced specificity and interactions with various neurotransmitter systems, leading to functional antagonism of the desired effect [8, 11, 14, 73, 74, 75, 76, 77].

As a natural polypharmacolgical agent, with evidence for its interaction with endocannabinoid and adenosine neuromodulating targets, we proposed that humulene would also interact with the GABAergic and serotonergic systems to elicit the behavioral response observed in *D. rerio*. Supporting our supposition, previous data pointed that the anxiolytic‐like effect of BCP in mice was mediated by the classical GABAergic B_ZD_ pathway [78], and Seekins et al. suggest that HUM would also affect the central dopamine and serotonin signaling to lead the antinociceptive behavior in mice [72]. Aligned with this, the third set of experiments was conducted to assess the possible involvement of GABA and 5‐HT targets in the mechanism of action of α‐humulene. The investigation proceeded with dose HUM‐20 justified by the absence of a statistical difference between its effect and that of the HUM‐40 dose and the D_ZP_ control, as well as the result of HUM‐40 in the open field test indicating a greater depressant potential on the animals' locomotor activity, which could lead to misleading interpretations.

### 2.4 GABAergic Neuromodulation

To investigate the anxiolytic mechanism of humulene via the GABA pathway, zebrafish were divided into experimental and control groups. The assayed group was treated with flumazenil (F_LZ_, GABA_A_R antagonist) followed by HUM‐20 (HUM + F_LZ_), with behavioral assessment in the light/dark test (0–300 s). Control groups received D_ZP_ + F_LZ_, D_ZP_ alone, F_LZ_ alone or DMSO_3%_.

**FIGURE 3:**
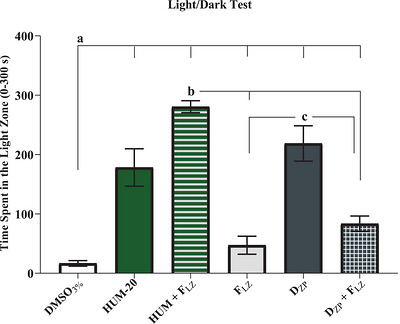
GABAergic neuromodulation of the anxiolytic‐like effect of α‐humulene in adult zebrafish in the light/dark test. Values are expressed as mean ± SEM; *n* = 6. Statistical analysis was performed using Welch's ANOVA followed by Games–Howell's post–hoc test. Letters indicate the order of statistically significant comparisons among groups. (a) DMSO_3%_ versus HUM‐20 (*p* < 0.05; *e* = 5); DMSO_3%_ versus HUM + F_LZ_ (*p* < 0.001; *e* = −1.51); DMSO_3%_ versus D_ZP_ (*p* < 0.01; *e* = 4.62); DMSO_3%_ versus D_ZP_ + F_LZ_ (*p* < 0.05; *e* = 5.36). (b) HUM + F_LZ_ versus F_LZ_ (*p* < 0.001; *e* = 17.75); HUM + F_LZ_ versus D_ZP_ + F_LZ_ (*p* < 0.001; *e* = 147.19). (c) D_ZP_ versus F_LZ_ (*p* < 0.01; *e* = 112.35); D_ZP_ versus D_ZP_ + F_LZ_ (*p* < 0.05; *e* = 110.54).

Figure 3 presents the results of GABAergic neuromodulation on the anxiolytic‐like effect of α‐humulene in adult zebrafish assessed in the light/dark test. Welch's ANOVA revealed a statistically significant difference among groups, F(5,13) = 100.4; *p* < 0.001; *ω* ^2^ = 0.784 (95% CI: 0.611 to 0.859). The Games–Howell post–hoc comparisons showed increased time in the bright zone for the treated groups compared to the negative control group, **a**: DMSO_3%_ versus HUM‐20 (Δ*M* = −161.50, 95% CI: −294.92 to −28.08; *p* < 0.05; *e* = 5); DMSO_3%_ versus HUM + F_LZ_ (Δ*M* = −264, 95% CI: −306 to −221.94; *p* < 0.001; *e* = −1.51); DMSO_3%_ versus D_ZP_ (Δ*M* = −202, 95% CI: −328.35 to −75.65; *p* < 0.01; *e* = 4.62); DMSO_3%_ versus D_ZP_ + F_LZ_ (Δ*M* = −66.83, 95% CI: −120.84 to −12.82; *p* < 0.05; *e* = 5.36).

The antagonist was able to reverse the action of the D_ZP_ control, but the same was not observed with humulene, **b**: HUM + F_LZ_ vs. F_LZ_ (Δ*M* = 233.50, 95% CI: 168.15 to 298.85; *p* < 0.001; *e* = 17.75); HUM + F_LZ_ versus D_ZP_ + F_LZ_ (Δ*M* = 197.17, 95% CI: 130.40 to 254.93; *p* < 0.001; *e* = 147.19). **c**: D_ZP_ versus F_LZ_ (Δ*M* = 171.50, 95% CI: 46.72 to 296.28; *p* < 0.01; *e* = 112.35); D_ZP_ versus D_ZP_ + F_LZ_ (Δ*M* = 135.17, 95% CI: 10.93 to 259.40; *p* < 0.05; *e* = 110.54).

Although there was no statistical difference between HUM‐20 and HUM + F_LZ_, the HUM + F_LZ_ group showed greater statistical significance than the isolated terpene dose when compared with the DMSO_3%_ control. This is corroborated by the significance of the HUM + F_LZ_ versus D_ZP_ + F_LZ_ comparison and also by the absence of a statistical difference between D_ZP_ and HUM + F_LZ_ (*p* > 0.4). These comparisons suggest an additive effect of F_LZ_ with the terpene.

GABA is the main inhibitory neurotransmitter of the central nervous system (CNS). Its GABA_A_R targets are widely distributed in the vertebrate CNS. They are transmembrane channels, formed by the union of five highly heterogeneous protein subunits (pentamers). In mammals, 19 subunits (*α* [1, 2, 3, 4, 5, 6], *β* [1, 2, 3], *γ* [1, 2, 3], *δ, ε, θ, φ, ρ* [1, 2, 3]) are recognized. This heterogeneity allows for the presence of various subpopulations of these channels, composed of distinct arrangements with specific topologies and sensitivities to other endogenous agents, drugs, and GABA itself. Zebrafish has 23 subunits (*α* [1; 2*a/b*; 3‐5; 6*a/b*]; *β* [1, 2, 3, 4], *γ* [1, 2, 3], *δ, φ, ζ, ρ* [1; 2*a/b*; 3*a/b*]) with spatial expressions and electrophysiological sensitivities to the neurotransmitter that suggest conserved properties. When activated by GABA, these channels allow the influx of chloride ions into neurons, leading to hyperpolarization and reduced excitability [79, 80].

Flumazenil's clinical function is to reverse the hypnotic and sedative effects of B_ZD_. Its antagonism is competitive and of high affinity for an allosteric site on GABA_A_Rs that contain the *γ*2 subunit. It physically occupies this target, preventing other ligands from modulating receptor activity. This blockade occurs with both positive allosteric modulators (PAM), which enhance the action of the endogenous agonist, and negative allosteric modulators (NAM), which reduce its action [81, 82, 83].

Previous studies have shown that GABAergic modulation by terpenoids is likely independent of the γ2 subunit presence in the GABA_A_R pentamer, and is also influenced by a cyclic molecular structure with oxygenated groups [36, 78]. Regarding the sesquiterpene and sesquiterpenoid subclasses, in addition to oxygenated functions in the ligand molecule, the presence of the γ2 or δ subunit in the channel appears to determine selectivity in the triggered response [36]. In *Xenopus laevis* oocytes containing channels formed by rat α1β2 subunits, BCP (bicyclic structure) caused a small boost in electrical current in response to GABA, while its derivative, caryophyllene oxide (bicyclic and oxygenated structure), had no influence. In human embryonic kidney cells (HEK‐293), which expressed a murine α1β2γ2 arrangement, characteristic of phasic (synaptic) GABA_A_Rs, these isomers were not relevant for the reinforcement or reduction of the response current [84].

Another study demonstrated that BCP had no action on channels formed by α1β2 subunits, while its hydroxylated derivative, β‐caryolanol, increased the electrical response current to GABA mediated by these channels. On channels with α4β3δ and α4β3δ arrangements, characteristic of tonic (extrasynaptic) GABA_A_R, these two agents reduced the response current to the neurotransmitter. However, in phasic channels, α1β2γ2, they did not reinforce or reduce the response current [36]. Interestingly, Oliveira demonstrated that F_LZ_ and bicuculline reversed the anxiolytic effect of orally administered BCP in mice, suggesting a PAM mechanism in phasic GABA_A_R channels containing the γ2 subunit [78].

In the study by Janzen et al. [36], humulol (a hydroxylated derivative of HUM) had complex effects. In HEK‐293 cells expressing α1β2 channels, this agent potentiated the maximum GABA response current, whereas in cells expressing recombinant phasic α1β2γ2 channels, it reduced it. In mouse hippocampal neurons (which naturally express GABA_A_Rs with various subunit arrangements), humulol again decrease the response current. This same effect was observed in those cells expressing channels with α4β3δ arrangement, thereby inferring a negative allosteric modulatory action. The authors reported that humulene had a negative allosteric modulator effect only on HEK‐293 expressing tonic α4β3δ channels, as it reduced the maximal electrical current response to GABA.

Tonic GABA_A_Rs (such as α4β3δ and α6β3δ) are located outside synapses; they are targets of neurosteroids and alcohol, and have high affinity and slow desensitization to GABA. This allows, even at low concentrations of this neurotransmitter in the extracellular environment, for the maintenance of basal inhibitory conductance independent of presynaptic action potentials. The induction of hyperpolarization by neurosteroids, with consequent reduction of neuronal excitability, is potent and capable of causing anesthesia. The loss of motility in zebrafish larvae caused by progesterone and glutamate‐pregnenolone, for example, may be a clue to the presence of channels with a tonic‐acting α4β3δ configuration conserved in this species. These receptors lack the classic B_ZD_ binding site and would not respond to F_LZ_ [81, 85, 86, 87, 88, 89].

However, Hanchar et al. showed the high‐affinity binding of the imidazo‐benzodiazepines [^3^H]RO15‐4513 and F_LZ_ to native bovine cerebellar α6βδ and to recombinant α4β3δ GABA_A_Rs expressed in HEK‐293T cells. Additionally, F_LZ_ competitively inhibited the binding of RO15‐4513, an alcohol antagonist, to α6βδ and α4β3δ, demonstrating that, unlike other classical B_ZD_ site ligands, it may act on GABA tonic receptors [90]. Further, Kuver and Smith presented that F_LZ_ is a negative allosteric modulator at the recombinant α4β2δ type, and prolonged exposure to this drug reduced the surface expression of these channels [91].

The additive effect of F_LZ_ on anxiolysis caused by HUM‐20 can be explained by the ability of α‐humulene to act as a negative allosteric modulator at tonic GABA receptors containing the δ subunit (*α*4β3δ), as demonstrated in vitro [36]. In this scenario, F_LZ_ would block this negative modulatory action, unmasking the primary anxiolytic effect. This interpretation is consistent with the high‐layer binding of F_LZ_ to receptors containing the δ subunit [90] and with the presence of such receptors in zebrafish [80]. The NAM action of humulene at tonic receptors may become predominant at higher doses, counterbalancing its main effect and explaining the non‐monotonic response profile presented. Furthermore, the classic B_ZD_ pathway can be ruled out as the primary mediator of humulene's motor effects, since F_LZ_ did not reverse its anxiolytic action in the light/dark test.

### 2.5 Serotonergic Neuromodulation

The investigation of the anxiolytic mechanism of humulene via the serotonin pathway was conducted by treating zebrafish with cyproheptadine (C_YPRO_, metabotropic serotonin type 2A receptor, 5‐HT_2A_R, antagonist, OG), pizotifen (P_ZTF_, a selective antagonist of the metabotropic serotonin type 1A and 2A/2C receptors, 5‐HT_1A_R, 5‐HT_2A/2C_R; OG), or granisetron (G_RAN_, 5‐HT_3_R antagonist, OG) before administering HUM‐20 (IP). These groups (HUM + C_YPRO_, HUM + P_ZTF_, and HUM + G_RAN_) were then assessed with the light/dark test (0–300 s). Controls included groups that received fluoxetine (F_LUOX_) plus antagonists (F_LUOX_ + C_YPRO_, F_LUOX_ + P_ZTF_, and F_LUOX_ + G_RAN_), only F_LUOX,_ C_YPRO_, P_ZTF_, G_RAN_, or DMSO_3%_.

**FIGURE 4:**
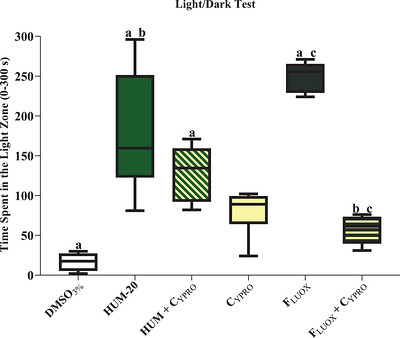
Action of C_YPRO_ on the anxiolytic‐like effect of α‐humulene in adult zebrafish in the light/dark Test. Statistical analysis was performed using Kruskal–Wallis test followed by Dunn's post–hoc test. Values are expressed as minimum, maximum, and percentiles (25th, 50th, and 75th); *n* = 6. DMSO_3%_ (Mdn = 17.50, IQR = 17.50); HUM‐20 (Mdn = 159.50, IQR = 83.50); HUM + C_YPRO_ (Mdn = 134.50, IQR = 47); C_YPRO_ (Mdn = 89, IQR = 18.50); F_LUOX_ (Mdn = 255.50, IQR = 24.75); F_LUOX_ + C_YPRO_ (Mdn = 61.50, IQR = 25). Letters indicate the order of statistically significant comparisons among groups. (a) DMSO_3%_ versus HUM‐20 (*p* < 0.001; rrb = 1.0); DMSO_3%_ versus F_LUOX_ (*p* < 0.001; rrb = 1.0); DMSO_3%_ versus HUM + C_YPRO_ (*p* < 0.01; rrb = 1.00); (b) HUM‐20 versus F_LUOX_ + C_YPRO_ (*p* < 0.01; rrb = −1.0); (c) F_LUOX_ versus F_LUOX_ + C_YPRO_ (*p* < 0.001; rrb = −1.0).

Figure 4 shows the results of C_YPRO_’s action on α‐humulene's anxiolytic‐like effect in zebrafish via the 5‐HT pathway. The Kruskal–Wallis test showed a statistically significant difference among groups, H(5) = 29.58; *p* < 0.001; *η* ^2^ = 0.819 (95% CI: 0.758 to 0.927). Dunn's post–hoc significant comparisons were **a**: DMSO_3%_ versus HUM‐20 (*z* = ‐3.683; *Wi* = 3.917 *Wj* = 26.33; *p* < 0.01; rrb = 1.0); DMSO_3%_ versus F_LUOX_ (*z* = −4.645; *Wi* = 3.917 *Wj* = 32.17; *p* < 0.001; rrb = 1.0), and DMSO_3%_ versus HUM + C_YPRO_ (*z* = −3.028; *Wi* = 3.917 *Wj* = 22.33; *p* < 0.01; rrb = 1.00); **b**: HUM‐20 versus F_LUOX_ + C_YPRO_ (*z* = 2.603; *Wi* = 26.33 *Wj* = 10.50; *p* < 0.01; rrb = −1.0); **c**: F_LUOX_ versus F_LUOX_ + C_YPRO_ (*z* = 3.563; *Wi* = 3.167 *Wj* = 10.50; *p* < 0.01; rrb = −1.0).

When associated with the F_LUOX_ control, C_YPRO_ caused a significant reduction in the anxiolytic parameter (*p* < 0.001), which is corroborated by previous studies with zebrafish model, where the presence of this antagonist reduced the effect of the F_LUOX_ control [65, 66, 67, 68, 69, 70, 71]. In this present, C_YPRO_ alone did not have a significant anxiolytic effect, which differs from those studies that demonstrated an anxiolytic effect of this drug alone administered to *D. rerio* [65, 66, 67, 68, 69, 70, 71]. The effect of humulene was not abolished by C_YPRO_ (HUM‐20 vs. HUM + C_YPRO_, *p* = 1), however, the graphic illustration shows that the presence of this antagonist reduced the time spent in the light zone. Even so, compared to the DMSO_3%_ control, the HUM + C_YPRO_ group remained anxiolytic with statistical significance (*p* < 0.05). This result indicate that the HUM mechanism of action does not directly involve metabotropic receptors 5‐HTR_2A_ but still suggest that its behavioral effect is mediated by 5‐HT targets. The further experiment using the antagonist P_ZTF_, selective for the 5‐HT_1A_R and 5‐HT_2A/2C_R subtypes, also confirm this initial result.

**FIGURE 5:**
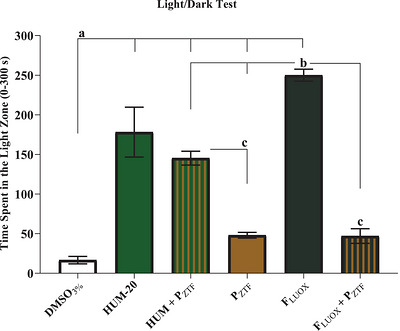
Action of P_ZTF_ on the anxiolytic‐like effect of α‐humulene in adult zebrafish in the light/dark test. Values are expressed as mean ± SEM, *n* = 6. Statistical analysis was performed using Welch's ANOVA followed by Games–Howell's post–hoc test. Letters indicate the order of statistically significant comparisons among groups. (a) DMSO_3%_ versus HUM‐20 (*p* < 0.05; *e* = 5); DMSO_3%_ versus HUM + P_ZTF_ (*p* < 0.001; e = 1.07); DMSO_3%_ versus P_ZTF_ (*p* < 0.01; *e* = 4.55); DMSO_3%_ versus F_LUOX_ (*p* < 0.001; *e* = −5.99). (b) F_LUOX_ versus HUM + P_ZTF_ (*p* < 0.001; *e* = 126.30); F_LUOX_ versus F_LUOX_ + P_ZTF_ (*p* < 0.001; *e* = 130.69); F_LUOX_ versus P_ZTF_ (*p* < 0.01; *e* = 125.82). (c) HUM + P_ZTF_ versus F_LUOX_ + P_ZTF_ (*p* < 0.001; *e* = 75.45); HUM + P_ZTF_ versus P_ZTF_ (*p* < 0.001; *e* = 71.51).

Figure 5 presents the results of the investigation of the action of P_ZTF_ on the anxiolytic‐like mechanism of α‐humulene in zebrafish via the 5‐HT pathway. Welch's ANOVA showed a statistically significant difference between groups, *F*(5,13) = 137.9; *p* < 0.001; *ω* ^2^ = 0.845 (95% CI: 0.720 to 0.900). The Games–Howell post–hoc comparisons were **a**: DMSO_3%_ versus HUM‐20 (Δ*M* = −161.50, 95% CI: −294.92 to −28.08; *p* < 0.05; *e* = 5); DMSO_3%_ versus HUM + P_ZTF_ (Δ*M* = −128.67, 95% CI: −165.45 to −91.88; *p* < 0.001; *e* = 1.07); DMSO_3%_ versus F_LUOX_ (Δ*M* = −233.33, 95% CI: −265.47 to −201.19; *p* < 0.001; *e* = −5.99).

P_ZTF_ was able to reduce the effect of F_LUOX_, **b**: F_LUOX_ versus F_LUOX_ + P_ZTF_ (Δ*M* = 203, 95% CI: 161.27 to 244.72; *p* < 0.001; *e* = 130.69); F_LUOX_ versus P_ZTF_ (Δ*M* = 202, 95% CI: 170.48 to 233.51; *p* < 0.01; *e* = 125.82). However, its action was less effective in the animals treated with humulene, **c**: HUM + P_ZTF_ versus F_LUOX_ + P_ZTF_ (Δ*M* = 98.33, 95% CI: 54.10 to 142.56; *p* < 0.001; *e* = 75.45); HUM + P_ZTF_ versus P_ZTF_ (Δ*M* = 97.33, 95% CI: 60.81 to 133.85; *p* < 0.001; *e* = 71.51).

The group treated only with the antagonist had a longer time spent in the light zone than the negative control, **a**: DMSO_3%_ versus P_ZTF_ (Δ*M* = −31.33, 95% CI: −52.20 to −10.45; *p* < 0.01; *e* = 4.55). However, this data does not point to an anxiolytic action, which is corroborated by previous studies that used this antagonist in *D. rerio* [65, 66, 67, 68, 69, 70, 71]. There was no statistical difference between F_LUOX_ and HUM‐20 (*p* > 0.3), also this positive control had greater significance when compared to the group that received the terpene plus the antagonist, **b**: F_LUOX_ versus HUM + P_ZTF_ (Δ*M* = 104.67, 95% CI: 64.27 to 145.05; *p* < 0.001; *e* = 126.30), however, it does not seem to reflect a main interaction of HUM with 5‐HT_1A_R and 5‐HT_2A/2C_R targets. Thus, it is inferred that α‐humulene does not act on 5‐HT classical metabotropic receptors and allows for a hypothesis of noncanonical interaction with the serotonergic system, possibly directed at fast signaling targets, such as the 5‐HT_3_R ionotropic channels. This hypothesis was tested in the experiment using G_RAN_, a selective 5‐HT_3_R blocker.

**FIGURE 6:**
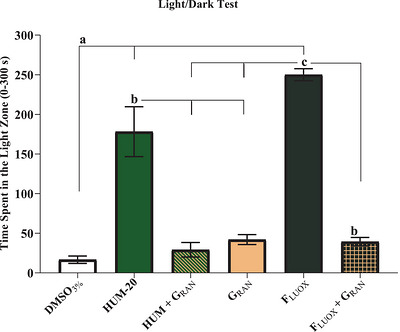
Action of G_RAN_ on the anxiolytic‐like effect of α‐humulene in adult zebrafish in the Light/Dark Test. Values are expressed as mean ± SEM, *n* = 6. Statistical analysis was performed using Welch's ANOVA followed by Games–Howell's post–hoc test. Letters indicate the order of statistically significant comparisons among groups. (a) DMSO_3%_ versus HUM‐20 (*p* < 0.05; *e* = 5.42); DMSO_3%_ versus F_LUOX_ (*p* < 0.001; *e* = −5.99). (b): HUM‐20 versus HUM + G_RAN_ (*p* < 0.05; *e* = 88.61); HUM‐20 versus G_RAN_ (*p* < 0.05; *e* = 88.23); HUM‐20 versus F_LUOX_ + G_RAN_ (*p* < 0.05; *e* = 86.97). (c) F_LUOX_ versus G_RAN_ (*p* < 0.001; *e* = 130.55); F_LUOX_ versus HUM + G_RAN_ (*p* < 0.001; *e* = 131.53); F_LUOX_ versus F_LUOX_ + G_RAN_ (*p* < 0.001; *e* = 129.77).

Figure 6 presents the results for the G_RAN_ action on the anxiolytic‐like mechanism of α‐humulene in zebrafish via the 5‐HT pathway. Welch's ANOVA showed a statistically significant difference between groups, F(5,13) = 128.5; *p* < 0.001; *ω* ^2^ = 0.863 (95% CI: 0.753 to 0.912). The Games–Howell post–hoc comparisons found that both treatments, HUM‐20 and F_LUOX_, increased time spent in the light zone versus the negative control group, **a**: DMSO_3%_ versus HUM‐20 (Δ*M* = −161.50, 95% CI: −294.92 to −28.08; *p* < 0.05; *e* = 5.42); DMSO_3%_ versus F_LUOX_ (Δ*M* = −233.33, 95% CI: −265.47 to −201.19; *p* < 0.001; *e* = −5.99).

G_RAN_ was effective in reversing the anxiolytic response of fish treated with both humulene and F_LUOX_, **b**: HUM‐20 versus G_RAN_ (Δ*M* = 136.16, 95% CI: 3.32 to 269; *p* < 0.05; *e* = 88.23); F_LUOX_ versus G_RAN_ (Δ*M* = 208, 95% CI: 173.71 to 242.28; *p* < 0.001; *e* = 130.55); HUM‐20 versus HUM + G_RAN_ (Δ*M* = 149, 95% CI: 17.23 to 280.77; *p* < 0.05; *e* = 88.61). **c**: F_LUOX_ versus F_LUOX_ + G_RAN_ (Δ*M* = 210.66, 95% CI: 177.75 to 243.57; *p* < 0.001; *e* = 129.77); HUM‐20 versus F_LUOX_ + G_RAN_ (Δ*M* = 138.83, 95% CI: 5.66 to 272; *p* < 0.05; *e* = 86.97); F_LUOX_ versus HUM + G_RAN_ (Δ*M* = 220.83, 95% CI: 179.43 to 262.23; *p* < 0.001; *e* = 131.53). There was no statistical difference in anxiety‐like behavior among the HUM + G_RAN_, F_LUOX_ + G_RAN_, and DMSO_3%_ control groups, nor between F_LUOX_ and HUM‐20.

The 5‐HT_3_ receptors are part of the Cys‐loop family of ligand‐gated ion channels, a group that also includes the nicotinic acetylcholine, GABA_A_, and glycine receptors. These channels are pentamers composed of the same monomer (3A) or different monomers (3A and 3B). Subunits 3A and 3B are identified in rodents and humans; notably, humans also have 3C–3E. In zebrafish, subunits A and B are present, and they are orthologous to the human A and B subunits. When serotonin binds, 5‐HT_3_R allows the passing of positive ions, such as sodium, potassium, and calcium—the incorporation of 3B subunit decreases the pentamer permeability to calcium—leading to rapid, transient excitation of the neuronal membrane. They are expressed in structures including the hippocampus, the frontal and entorhinal cortices, the amygdala, and the nucleus accumbens. The distribution of 5‐HT_3_R in these regions, therefore, serves as a predictive reference for their pharmacological manipulation in behavioral assays. Furthermore, about 80% of these receptors are located on synaptic terminals, where their activation can modulate the release of dopamine, norepinephrine, GABA, acetylcholine, glutamate, substance P, cholecystokinin, and 5‐HT itself [92, 93, 94, 95, 96, 97].

Granisetron acts competitively and selectively at the orthosteric site of the 5‐HT_3_ channel, preventing 5‐HT from physically occupying the site and blocking its excitatory actions. Its expected clinical effect is to prevent hyperstimulation of specific excitatory pathways, particularly in conditions of elevated serotonergic tone, such as emesis and vomiting caused by chemotherapy [93]. In presynaptic neurons, 5‐HT_3_R antagonism tends to suppress dopaminergic transmission and increase depressive symptoms. Its activation results in the facilitation of GABA release and a reduction in anxiety‐like behaviors, an effect that is counteracted by agents like G_RAN_. Therefore, the inhibition of these receptors on the presynaptic membrane may produce anxious and depressive phenotypes [93].

Nowicki et al. demonstrated that zebrafish treated with ondansetron, the prototype of 5‐HT_3_R antagonist class, increased bottom‐dwelling in the novel tank test, a behavior interpreted as anxiety [98]. In the rodent model literature, the 5‐HT_3_R antagonism has shown promising anxiolytic and antidepressant effects, but also anxiogenic effects. This does not support the inference of an isolated role for 5‐HT and 5‐HT_3_R in modulating these states, but rather suggests a circuit‐dependent action. For example, increased 5‐HT activity in the amygdala increases the expression of anxious behaviors, whereas in the periaqueductal gray matter, it has a panicolytic effect [93, 94, 99].

Most 5‐HT_3_R antagonists derived from natural sources lack structural similarity to serotonin. Among the structurally related phytoconstituents that modulate voltage‐gated and/or ligand‐gated ion channels, terpenoids (oxygenated terpenes) such as menthol, citral, geraniol, and eucalyptol inhibit the maximal 5‐HT response in a concentration‐dependent manner and do not compete with G_RAN_ for the orthosteric site. Modeling assays predict that these ligands bind in a transmembrane cavity at the interface between adjacent subunits, a phenomenon probably explained by their lipophilicity [92, 93, 100]. This suggests that oxygenated functional groups (hydroxyl, ketone, aldehyde, and ester, among others) in this class of natural molecules are necessary for their interaction with Cys‐loop family ion channels.

Other lipophilic and oxygenated natural agents, similar to terpenoids, inferred as NAM at 5‐HT_3_R, are the cannabinoids Δ9‐tetrahydrocannabinol (exogenous) and anandamide (endogenous). In the results of Barann et al., these components inhibited the 5‐HT response current in HEK‐293 cells expressing recombinant human 5‐HT_3_R. This effect was not blocked by rimonabant, antagonist of the CB1 receptor, which allowed an inference about a modulatory action at an allosteric target site in these channels [101].

The work of LaVigne et al. revealed the cannabimimetic action of HUM in mice, suggesting low‐potency agonism or positive modulation at CB1, as well as modulatory effects on plasma membrane dynamics (attributed to its lipophilicity) in regard to affect the synthesis or degradation of endogen cannabinoids [35]. In zebrafish, this neuromodulator system has expression profiles similar to those in mammals. Its receptor targets can be detected as early as the embryonic stage in this species and CB1 shares 70% protein sequence identity with its human ortholog [39, 102]. Therefore, it is possible that α‐humulene shares with other cannabinoids a modulatory feature on 5‐HT_3_R which leads to the behavioral response observed in *D. rerio*.

To further support these results and the hypothesis for the α‐humulene anxiolytic‐like action mechanism, a molecular docking protocol was conducted to evaluate the possible interactions of this sesquiterpene with the human GABA_A_R and the mouse 5‐HT_3A_R. The findings are presented below.

### 2.6 Molecular Docking

**FIGURE 7:**
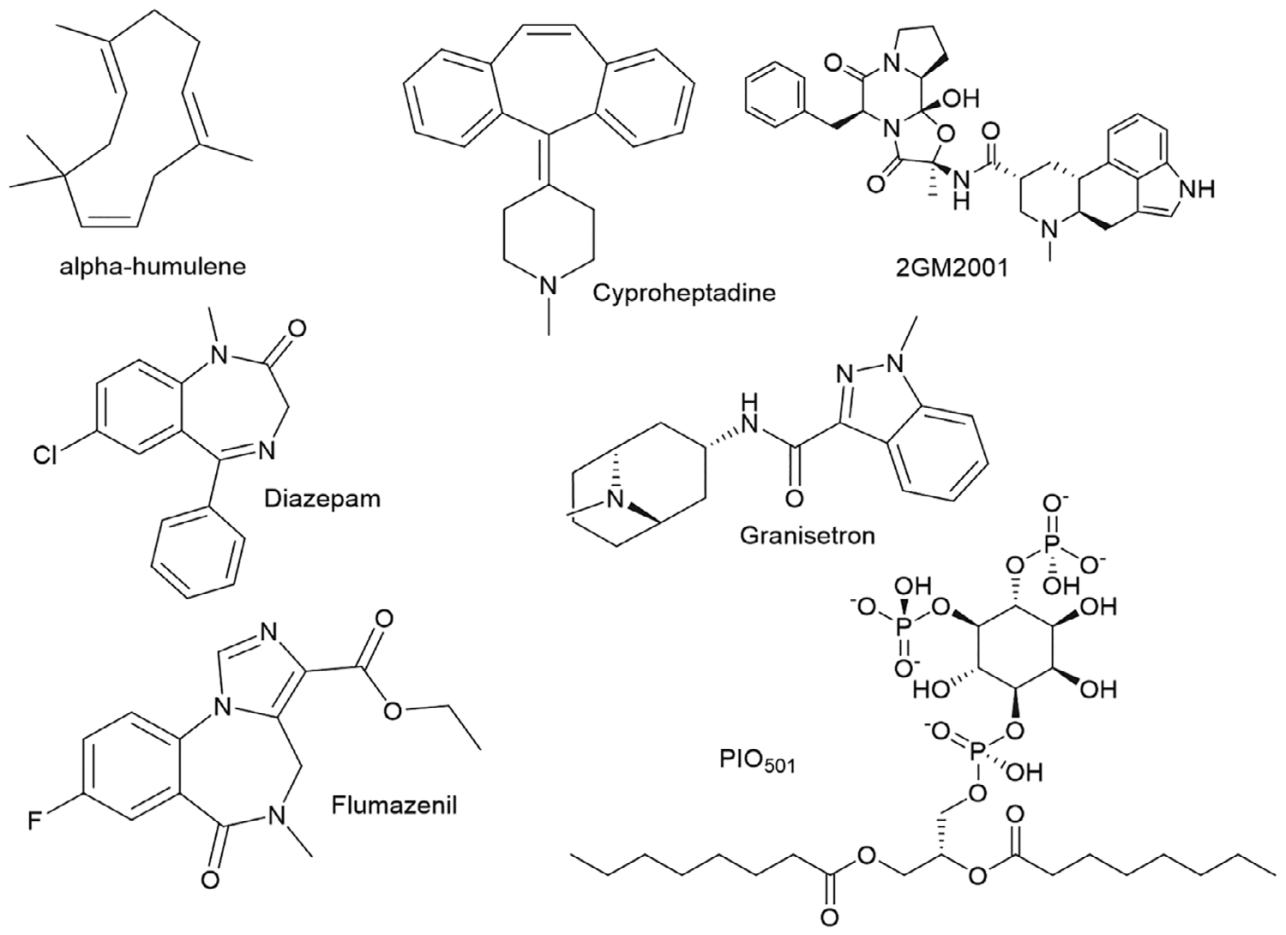
Chemical structure draw of α‐humulene (tested ligand) and the reference ligands: D_ZP_, F_LZ_ and PIO501 (GABA_A_R); C_YPRO_, 2GM2001, and G_RAN_ (5‐HT_3A_R). Made by the MarvinSketch code, and saved at physiological pH.

All docking and redocking simulations yielded root mean square deviation (RMSD) values less than or equal to the established reference threshold of 2.0 Å [103], demonstrating the robustness and internal consistency of the computational protocol employed (Table 1). For the GABA_A_ receptor, the analysis revealed RMSD values of 0.14 Å (PIO501/GABA_A_R), 1.38 Å (F_LZ_/GABA_A_R), 1.41 Å (α‐humulene/GABA_A_R), and 2.00 Å (D_ZP_/GABA_A_R), indicating excellent reproducibility of the crystallographic binding modes across distinct ligand classes. With respect to the 5‐HT_3A_ receptor, all tested ligands (Figure 7) exhibited RMSD values of 2.0 Å, which is fully acceptable within the methodological cutoff and further supports the reliability of the docking strategy. Collectively, these results confirm that the selected parameters and scoring procedures were adequate to reproduce experimentally observed poses, thereby providing a solid basis for subsequent interaction and affinity analyses.

**TABLE 1:** Initial result of the molecular docking simulation on the human GABA_A_ and mouse 5‐HT_3A_ receptors.

Ligands	Human GABA_A_ receptors		Mouse 5‐HT_3A_ receptors	
	Energy affinity (kcal/mol)	RMSD (Å)	Energy affinity (kcal/mol)	RMSD (Å)
α‐Humulene	Pose 9/−5.1	1.41	Pose 1/−7.8	2.00
PIO501	Pose 8/−5.1	0.14	—	—
Diazepam	Pose 2/−6.5	2.00	—	—
Flumazenil	Pose 2/−5.6	1.38	—	—
2GM2001	—	—	Pose 2/−10.8	2.00
Cyproheptadine	—	—	Pose 1/−9.7	2.00
Granisetron	—	—	Pose 1/−9.2	2.00

### 2.7 Interactions of Targets

The docking analyses conducted for both the GABA_A_ and 5‐HT_3A_ receptors provided mechanistic insights into the binding characteristics of α‐humulene relative to well‐established pharmacological standards. The redocking validation confirmed the reliability of the computational protocol, as the co‐crystallized ligands PIO501 (GABA_A_R) and 2GM2001 (5‐HT_3A_R) reproduced their crystallographic poses with RMSD values within the accepted threshold of 2.0 Å, supporting the robustness of the subsequent simulations.

### 2.8 Interactions at the Human GABA_A_ Receptor

**FIGURE 8:**
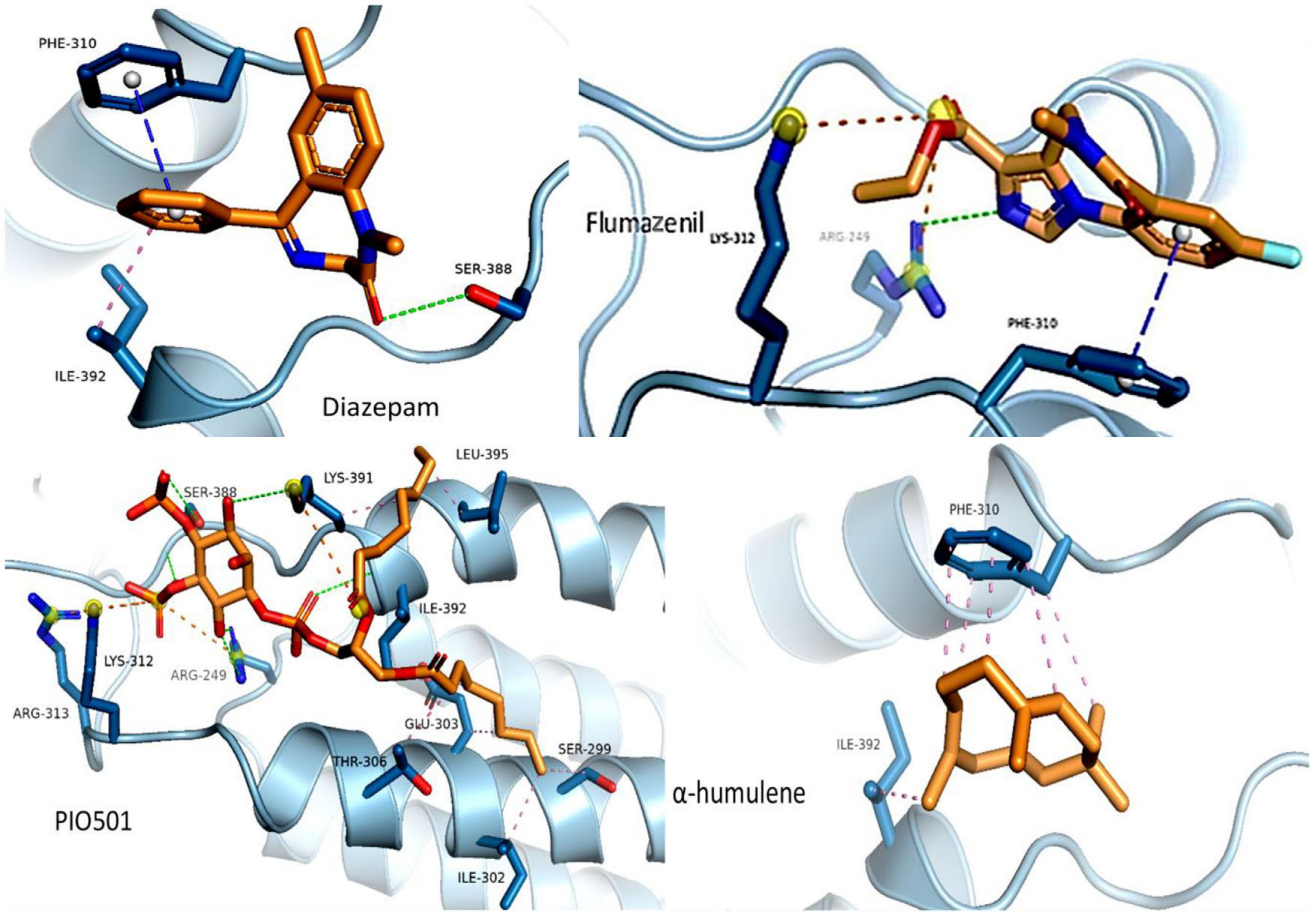
Predicted binding poses of α‐humulene and reference ligands in the human GABA_A_ receptor. D_ZP_ (upper left) and F_LZ_ (upper right) exhibit the expected π–π stacking interactions with PHE 310 and polar contacts with SER 388, LYS 312, and ARG 249, characteristic of high‐affinity B_ZD_ modulators. The co‐crystallized ligand PIO501 (lower left) establishes the canonical hydrogen‐bond and electrostatic network that defines the B_ZD_ allosteric pocket. In contrast, α‐humulene (lower right) presents a hydrophobic anchoring pattern involving PHE 310 and ILE 392, consistent with low‐polarity sesquiterpene modulators. Hydrogen bonds, hydrophobic interactions, and aromatic interactions are indicated by green, purple, and orange dashed lines, respectively.

The standard B_ZD_, D_ZP_, displayed the characteristic interaction triad at the B_ZD_ site—π–π stacking with phenylalanine (PHE 310), polar stabilization through serine (SER 388), and hydrophobic support from isoleucine (ILE 392)—closely matching crystallographic observations [104]. F_LZ_, used as the inverse/neutral modulator reference, maintained anchoring interactions with PHE 310 and lysine (LYS 312), indicative of its antagonistic profile. The co‐crystallized ligand PIO501, used for redocking validation, engaged with arginine (ARG 249), SER 388, LYS 312, and glutamate (GLU 303), forming a dense network that defines the canonical boundaries of the B_ZD_ allosteric binding site (Figure 8).

Relative to the standards, α‐humulene exhibited a significantly hydrophobic interaction profile, interacting mainly with PHE 310 and ILE 392, without forming the characteristic hydrogen bonds observed for D_ZP_ and PIO501 (Figure 8). Similarly, Janzen et al. also reported in silico the purely hydrophobic interaction of HUM at the D_ZP_ binding site in GABAR_A_ [36]. This pattern is consistent with compounds that act as low‐affinity modulators, relying primarily on hydrophobic complementarity rather than classical B_ZD_ pharmacophores. Such profile is coherent with mild anxiolytic or sedative‐like activities reported for sesquiterpenes in vivo [105, 106]. Thus, if α‐humulene can positively modulate GABA ionotropic channels (such as phasic GABA_A_Rs) out of the B_ZD_‐binding site, this may explain the failing of F_LZ_ to reverse its effect. However, this low‐affinity mechanism does not seem to be the major promoter of anxiolysis, yet it allows us to infer a contributor to a relaxant and/or sedative effect in vivo, as it would enhance the GABAergic inhibition.

### 2.9 Interactions at the Mouse 5‐HT_3A_ Receptor

**FIGURE 9:**
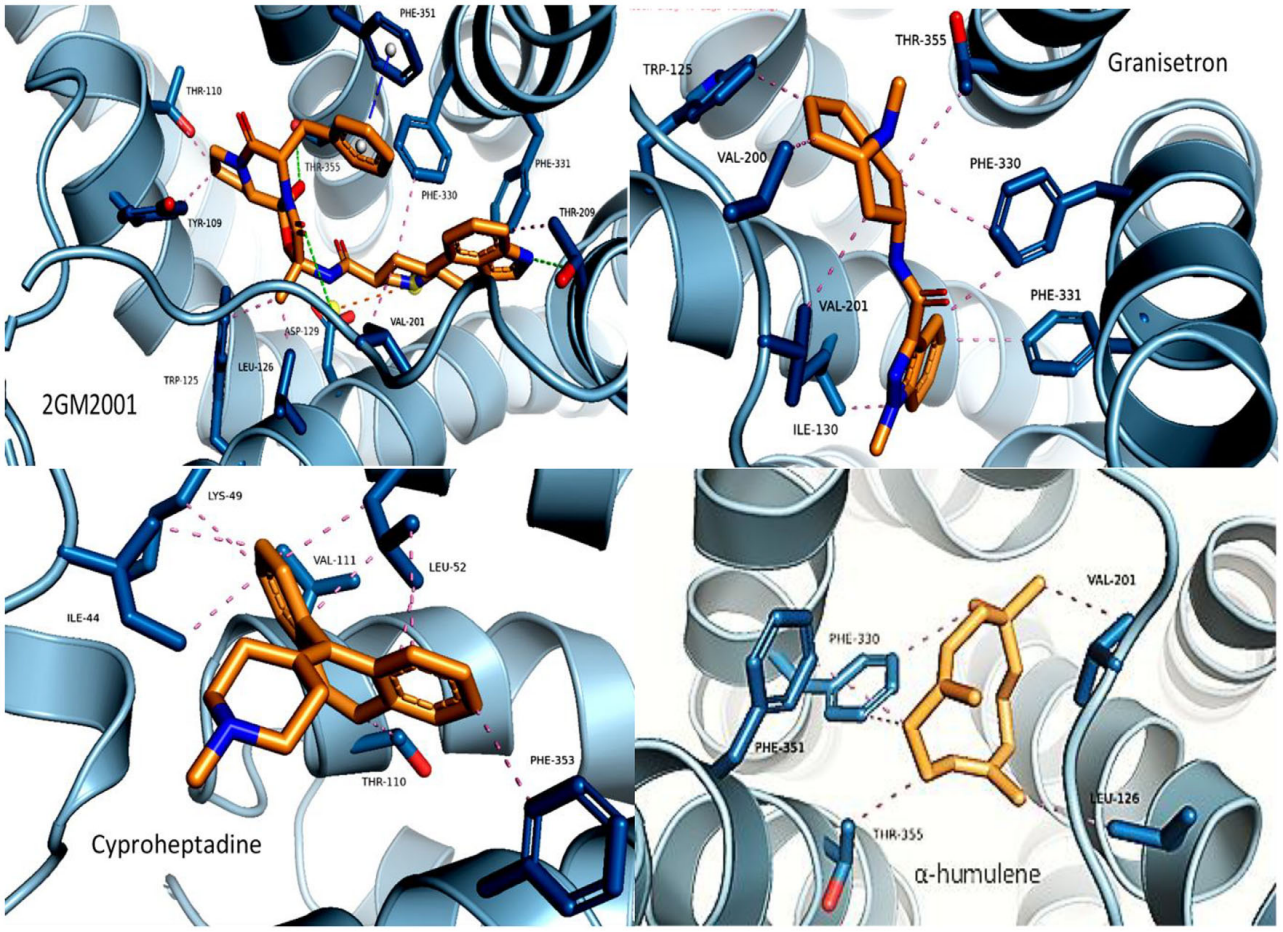
Predicted binding poses of α‐humulene and reference ligands in the mouse 5‐HT_3A_ receptor. The co‐crystallized ligand 2GM2001 (upper left) demonstrates the canonical interaction network with TYR 109, TRP 125, PHE 330, 331, and 351. The docking poses of G_RAN_ (upper right), C_YPRO_ (lower left), and α‐humulene (lower right) reveal distinct interaction profiles, with α‐humulene engaging predominantly hydrophobic residues (e.g., VAL 201, LEU 126, and PHE 330), whereas the reference antagonists display π–π stacking and polar contacts consistent with classical orthosteric recognition. Hydrogen bonds, hydrophobic interactions, and π–π interactions are represented by green, purple, and orange dashed lines, respectively.

The orthosteric reference ligand 2GM2001 exhibited the expected network of interactions with tyrosine (TYR 109), tryptophan (TRP 125), phenylalanine (PHE 330, 331, and 351), consistent with structural data available for the 5‐HT_3_ receptor family [107, 108]. G_RAN_, a clinically validated antagonist, showed strong cation‐π and π–π interactions within this aromatic‐rich pocket, reinforcing its efficient orthosteric blockade, as widely reported in the literature. C_YPRO_, although a nonselective antagonist, demonstrated a more hydrophobic signature within the binding cavity, engaging with residues such as valine (VAL 111), leucine (LEU 52), and threonine (THR 110) (Figure 9), which is compatible with its broader receptor profile and less rigid orthosteric anchoring [109].

In contrast, α‐humulene occupied predominantly hydrophobic regions of the cavity and interacted with PHE 330 and 351, VAL 201, and LEU 126 through Van der Waals forces. The absence of polar or cationic interactions, in addition to its incomplete overlap with the G_RAN_/2GM2001 orthosteric region, strongly suggests a nonclassical modulatory binding mode (Figure 9). Similar hydrophobic terpene interactions have been associated with mild attenuation of serotonergic signaling and behavioral effects in zebrafish models [110, 111]. Thus, the docking data support the hypothesis that α‐humulene modulates 5‐HT_3_R function allosterically or semi‐orthosterically, rather than acting as a canonical agonist or competitive antagonist.

### 2.10 Comparative Interpretation and Mechanistic Implications

Collectively, the docking results reveal that the reference ligands—G_RAN_ and C_YPRO_ for 5‐HT_3A_R, and D_ZP_, F_LZ_, and PIO501 for phasic GABA_A_R—exhibit patterns of high‐affinity orthosteric or allosteric binding. Their interaction profiles serve as robust benchmarks against which α‐humulene can be evaluated.

In both receptors, α‐humulene consistently adopted hydrophobic and non‐orthosteric poses, suggesting a mechanism of action based on allosteric modulation rather than competitive binding. This type of receptor engagement is typical for lipophilic terpenoids lacking polar substituents. It has been associated with neuromodulatory effects mediated through subtle conformational adjustments rather than direct agonism or antagonism [112]. These findings provide molecular rationale for the in vivo outcomes. The joint modulation of GABAergic and serotonergic pathways, both through hydrophobic allosteric contacts, may account for the observed responses of adult zebrafish Thus, the docking results not only corroborate the behavioral profile but clarify the distinct mechanistic differences between HUM and the standard ligands used in this study.

### 2.11 Integrated Discussion

Alpha‐humulene induces an anxiolytic‐like effect in adult zebrafish. The results suggest a non‐monotonic dose‐response profile, which was not blocked by F_LZ_, but it was completely abolished by the antagonist G_RAN_, demonstrating a dependent mechanism on the fast 5‐HT neurotransmission (Table 2). This contrasts with previous findings for its isomer, BCP, which had its anxiolytic effect reversed by F_LZ_, but was not abolished by ondansetron [78].

**TABLE 2:** Summary of pharmacological blockade experiments for hum‐20 anxiolytic‐like effect in adult zebrafish.

Target pathways	Antagonists used	Predicted results if involved	Observed results	Conclusion
GABA_A_R—B_ZD_ Site	Flumazenil	Reversal of effect	No reversal**;** inferred secondary mechanism of negative allosteric modulation at tonic GABA_A_R; (Figure 3).	Independent
5‐HT_2_ Receptors	Cyproheptadine	Reversal of effect	No reversal; (Figure 4).	Independent
5‐HT_1A_, 5‐HT_2A/2C_ Receptors	Pizotifen	Reversal of effect	No reversal; (Figure 5).	Independent
5‐HT_3_ Receptor	Granisetron	Reversal of effect	Complete reversal; inferred I. Positive allosteric modulation at 5‐HT_3_R or II. A subsequent recruitment of the 5‐HT_3_R signaling (Figure 6)	Dependent

To test the hypothesis that humulene would act similarly to classic anxiolytics like D_ZP_, zebrafish were pretreated with F_LZ_, a competitive antagonist that selectively blocks the B_ZD_ allosteric binding site on GABA_A_R. F_LZ_ failed to reverse the anxiolytic effect of HUM‐20. The HUM + F_LZ_ group remained highly anxiolytic, showing no statistical difference from the HUM‐20 group alone. The experiment was validated, as F_LZ_ pretreatment successfully reversed the anxiolytic effect of the positive control D_ZP_. Interestingly, the data suggest a subtle, additive or potentiating effect, as the HUM + F_LZ_ group showed greater statistical significance (versus the DMSO_3%_ control) than HUM‐20 alone. This may be caused by the HUM allosteric negative modulation at a subpopulation of tonic GABA_A_Rs (previous in vitro reports), which would become functionally dominant, counteracting its own primary effect at higher doses, and further provides an explanation for the *D. rerio* behavioral non‐monotonic dose‐response to humulene.

Although HUM promoted a monotonic dose‐dependent reduction in the zebrafish locomotor activity in the open field test, an effect shared by the positive control D_ZP_, the subsequent demonstration of HUM‐20 anxiolysis in the presence of F_LZ_ may distinguish that open field result from the B_ZD_‐sedation phenotype. Otherwise, it suggests an adverse cataleptic‐like motor suppression, which is corroborated by previous observations in mice, where humulene produced cataleptic behavior mediated by A2A and CB1 receptors [35].

Having excluded the canonical GABA pathway, the investigation turned to the next major anxiety circuit: the serotonergic system. Pretreatment with C_YPRO_ (a broad 5‐HT_2_R antagonist) and P_ZTF_ (a 5‐HT_1A_R and 5‐HT_2A/2C_R antagonist) failed to reverse the anxiolytic effect of HUM‐20, what allowed us to rule out the direct involvement of the metabotropic 5‐HT_1A_, 5‐HT_2A/2C_ receptors. Pretreatment with G_RAN_, a selective 5‐HT_3_R antagonist, completely abolished the anxiolytic‐like effect of HUM‐20. The behavior of the HUM‐20 + G_RAN_ group was not statistically different to the negative control (DMSO_3%_) group. This result pinpoints the HUM's mechanism to a specific excitatory pathway, as the 5‐HT_3_ receptors, ligand‐gated ionic channels, are part of the Cys‐loop family (along with GABA_A_Rs) and mediate rapid, transient excitation in neuronal membranes.

Despite lacking oxygenated functions in its monocyclic molecule, HUM is lipophilic, which may favor interaction with hydrophobic sites of 5‐HT_3_R within the plasma membrane. One proposition for its direct action is that α‐humulene is a positive allosteric modulator at those channels. As a PAM, it would not activate the receptor itself but would enhance the effect of endogenous serotonin, consistent with anxiolysis. As a competitive antagonist at the orthosteric site, G_RAN_ would block the anxiolytic effect by preventing the primary binding of 5‐HT to the channel, which is necessary for HUM's proper function.

Accordingly, in silico analyses revealed plausible interactions of this terpene with hydrophobic transmembrane regions, consistent with noncanonical modulation of ionotropic receptors. Together with behavioral pharmacology, these findings corroborate that α‐humulene may act through an allosteric mechanism that enhances fast serotonergic signaling via 5‐HT_3_R. In the rodent and nonhuman primate adult forebrain 5‐HT_3_R is predominantly expressed in subpopulations of GABAergic interneurons that do not express parvalbumin and/or somatostatin [113, 114, 115, 116, 117, 118, 119, 120]. In the zebrafish telencephalon, the expression of GABAergic interneurons points to the evolutionary conservation of neuromodulation mechanisms with other vertebrate groups, also including the presence of genes for 5‐HT_3_R channels [121, 122]. Thus, we hypothesize that the α‐humulene effect observed in *D. rerio* may be directly mediated by 5‐HT_3_R, which promotes the activation of telencephalic GABA‐releasing neurons to inhibit excitatory neuron cells that elicit aversive‐fear behavior.

In line with previous reports, another possible interpretation is that the humulene anxiolytic‐like effect would be primarily mediated by endocannabinoid and adenosine targets [34, 35]. We hypothesize that α‐humulene interaction on both endocannabinoid CB1 and adenosine A2A receptors promotes subsequent 5‐HT_3_R fast signaling in circuits involved in the zebrafish scototactic avoidance. The A2A component may directly facilitates the local 5‐HT release in *D. rerio* telencephalon [123, 124, 125], while the CB1 component disinhibits serotonergic raphe nuclei by reducing the inhibitory tone of GABA [126, 127, 128, 129, 130, 131]. The augmented serotonin phasic surge targets the ionotropic 5‐HT_3_ channels expressed on local GABAergic interneurons, which rapidly cause neuronal depolarization and a consequent localized release of GABA. This final GABA output would inhibit excitatory cells in anxiety‐driving circuits, resulting in the anxiolytic‐like behavioral response. Although this hypothesis is plausible for mammals, supported by previous evidence in rodent models [34, 35], the present study did not directly investigate this possibility for the zebrafish model. Future experiments employing selective antagonists, such as rimonabant for CB1 and SCH58261 for A2A, may elucidate whether α‐humulene acts primarily on the endocannabinoid and/or adenosinergic targets to unleash an upstream cascade that recruits the serotonergic fast signaling or as a direct modulator of the 5‐HT_3_ receptor.

## 3 Conclusions

This study demonstrates that the sesquiterpene α‐humulene did not exhibit acute toxicity and induced anxiolytic‐like behavior in adult zebrafish. Mechanistic investigation revealed a pharmacology independent of the B_ZD_ binding site on the GABA type A channel. This effect was completely reversed by G_RAN_, implicating rapid serotonergic signaling as a crucial component of the resulting behavior. The convergence of behavioral, mechanistic, and computational evidence positions this sesquiterpene as a promising natural product for modulating mood and anxiety‐related behavior via nonclassical serotonin target. Furthermore, the low acute toxicity and absence of a positive interaction at the classical B_ZD_ site suggest that humulene, and possibly its derivatives, could be investigated as prototypes for a new class of anxiolytics with less sedative side effects. Future studies should evaluate chronic toxicity, refine pharmacokinetic and behavioral parameters, and employ selective antagonists of A2A and CB1 receptors, as well as other 5‐HT receptor subtypes, including neurochemical measurements to fully unravel direct circuit‐level interactions and corroborate the mechanistic insights provided by our pharmacological and in silico approaches.

## 4 Experimental Section

### 4.1 Drugs

The following substances were used: α‐humulene (Sigma–Aldrich, 96% ≥ pure), D_ZP_ (NeoQuímica), DMSO (Dynamic), F_LZ_ (Sandoz), F_LUOX_ (Teuto), C_YPRO_ (Cobavital), P_ZTF_ (Sandomigran), and G_RAN_ (Kytril) (Table 3).

**TABLE 3:** Summary of pharmacological agents, doses, and administration protocols.

Compounds	Doses (mg kg^−1^)	Vehicles	Administration routes	Volume (µL)	Timing
Alpha‐humulene	4, 20, 40	DMSO_3%_	Intraperitoneal	20	30 min pretest
Dimethyl sulfoxide (DMSO)	Solution of 3% DMSO (DMSO_3%_)	N/A	Intraperitoneal	10	30 min pretest
Diazepam (D_ZP_)	4	N/A	Intraperitoneal	10	30 min pretest
Fluoxetine (F_LUOX_)	0.05	N/A	Intraperitoneal	10	30 min pretest
Flumazenil (F_LZ_)	0.05	N/A	Intraperitoneal	10	45 min pretest
Cyproheptadine (C_YPRO_)	32	N/A	Oral gavage	10	45 min pretest
Pizotifen (P_ZTF_)	32	N/A	Oral gavage	10	45 min pretest
Granisetron (G_RAN_)	20	N/A	Oral gavage	10	45 min pretest

### 4.2 Animals

Zebrafish (aged 90–120 days; 0.4 ± 0.1 g; 3.5 ± 0.5 cm) of the wild strain, of both sexes, were acquired from a local aquarium store (Fortaleza, Ceará, Brazil). The animals were maintained in a 10 L glass tank (30 × 15 × 20 cm) (stocking density of five fish per liter), with dechlorinated water (ProtecPlus) and external hang‐on filters, at a temperature of 28°C, pH 7.0, and a 14 h / 10 h (light / dark) circadian cycle. The fish received feed (Alcon Basic) *ad libitum* until 24 h before the experiments.

The prior sample size calculus was performed using GPower 3.1.9.7 software for *F*‐tests (One‐way ANOVA) [132, 133, 134]. The values for effect size (*f*), alpha (*α*), power (assuming *β* = 0.2), and groups (N) were 0.5, 0.05, 0.8, and 18, respectively. Thus, the total sample size suggested was 108 for the behavioral tests. In order to comply with the 3Rs principles, the groups of fish assessed in the open field test were the same used in the light/dark test. Additionally, the control groups and the chosen anxiolytic dose values were used for comparisons in the pharmacological dissection set of experiments. The study was approved by the Ethics Committee on the Use of Animals of the State University of Ceará (CEUA‐UECE; n° 04983945/2023), in accordance with the Ethical Principles of Animal Research.

### 4.3 General Protocol

The experiments occurred on three occasions. First, acute toxicity was assessed to determine the safety of humulene at three doses chosen for the experiments. Second, once nontoxic features were observed, these same doses were used in the open field followed by the light/dark tests to evaluate its effects on the zebrafish behavioral response. Third, a single dose of humulene (which previously showed better anxiolytic efficacy in the light/dark test) was combined with pharmacological antagonists in a repeated light/dark test to investigate its mechanism of action. Adult zebrafish of both sexes were randomly selected and allocated between the groups, ensuring balanced distribution among all experimental groups. Each group set for experimentation had a number of six animals, and all behavioral assessments were performed blindly by the experimenters.

The handling was conducted by first placing each fish in a container filled with cold water (10°C–12°C) for one to three minutes until it reached a state of reduced opercular movements and swimming activity. Then, it was transferred to a moist sponge and treated, whether by IP injection or OG. A micropipette was used for the OG. The sesquiterpene was diluted in a vehicle solution of DMSO_3%_, and the doses (4, 20, or 40 mg kg^−^^1^) were injected intraperitoneally at a volume of 20 microliters by an insulin syringe (0.5 mL; UltraFine BD) with a 30G gauge needle. The DMSO_3%_ concentration was selected based on established protocols in the literature for IP administration in adult zebrafish, and is widely used as a vehicle for lipophilic compounds in behavioral assays with this species [135, 136, 137, 138]. The other drugs used were diluted only in distilled water. Immediately after treatment, the fish were placed in a beaker (250 mL) filled with home aquarium water (150 mL) at 28°C for recovery to follow the behavioral assessment. Soon after finishing the experiments, fish were euthanized by immersion in ice‐chilled water (0°C–2°C) for 10 min [63, 64, 65, 66, 67, 68, 69, 70, 71, 139].

### 4.4 Acute Toxicity Evaluation

Fishes (*n* = 6) were treated intraperitoneally with HUM‐4, HUM‐20, or HUM‐40. After treatments, the animals were left undisturbed for 96 h to assess mortality. The number of dead fish in each group was recorded every 24 h. The lethal dose capable of killing 50% of the animals (LD_50_) was determined by the Trimmed Spearman–Karber mathematical method, with a 95% confidence interval [140].

### 4.5 Locomotor Activity Assessment

Groups (*n* = 6) were treated with HUM‐4, HUM‐20, HUM‐40, D_ZP_ (4 mg kg^−1^; 10 µl, IP, positive control), or DMSO_3%_ (10 µL, IP, negative control). After 30 min of treatment, the animals were individually placed in Petri dishes (10 × 15 cm; with quadrants on the bottom), containing the same water from their home tank. The number of line crossings was recorded (0–300 s) [63, 64, 65, 66, 67, 68, 69, 70, 71].

### 4.6 Anxiolytic‐Like Effect Assessment

For this protocol, a glass tank (30 × 15 × 20 cm) was divided into a bright area and a dark area. It was filled up to reach a 3 cm water (dechlorinated tap water) column. This novel and shallow environment, different from the conventional home tank, is capable of inducing anxiety‐like behavior.

The tested doses of humulene, control groups, and treatment routes were the same as previously described in the locomotor activity assessment protocol. The animals were individually placed in the light/dark tank, and the anxiolytic effect was evaluated by recording the time that each fish remained in the bright zone of the tank during the 5 min test period [63, 64, 65, 66, 67, 68, 69, 70, 71].

### 4.7 GABAergic Neuromodulation

The investigation of the possible involvement of the GABAergic pathway in the anxiolytic‐like effect of humulene was performed by pre‐treating the animals with F_LZ_ (0.05 mg kg^−1^, 10 µL, IP) before the light/dark test. The fishes (*n* = 6) received F_LZ_ 15 min before the administration of the most effective anxiolytic dose of humulene (HUM‐20; 20 µL; IP) identified in the previous light/dark test. Besides the groups DMSO_3%_ alone and D_ZP_ alone, a group that received only F_LZ_ and another that received D_ZP_ plus F_LZ_ were included as controls. After 30 min of the treatments, the animals were subjected to the light/dark test for behavioral assessment [63, 64, 65, 66, 67, 68, 69, 70, 71].

### 4.8 Serotonergic Neuromodulation

To investigate the possible involvement of the serotonergic pathway in the anxiolytic‐like effect of humulene, groups (*n* = 6) were treated with C_YPRO_ (32 mg kg^−1^, 10 µL, OG), P_ZTF_ (32 mg kg^−1^, 10 µL, OG), or G_RAN_ (20 mg kg^−1^, 10 µL, OG) 15 min before injection of HUM‐20 (20 µL, IP). The control groups were DMSO_3%_; F_LUOX_ (0.05 mg kg^−1^, 10 µL, IP); C_YPRO_; P_ZTF_; G_RAN_; F_LUOX_ plus C_YPRO_; F_LUOX_ plus P_ZTF_; or F_LUOX_ plus G_RAN_. After 30 min of the treatments, the animals were subjected to the light/dark test for behavioral assessment (0–300 s) [63, 64, 65, 66, 67, 68, 69, 70, 71].

## 5 Molecular Docking

### 5.1 Computational Codes

To carry out the simulations, the codes used were: MarvinSketch 19.12.0 [141], Avogadro [142], Autodocktools [143], AutoDockVina [144], UCSF Chimera [145], Discovery studio visualizer viewer [146], and Pymol [147].

### 5.2 Ligand Design and Optimization

The chemical structure of the tested ligand and the standard ligands of each receptor (GABA_A_R and 5‐HT_3A_R) studied in the simulation were drawn using the MarvinSketch code [141], saved at physiological pH (Figure 7), and the lowest energy conformers were optimized using the Avogadro code [142], configured to use steepest descent algorithm with cycles of 50 iterations, applying the MMFF94 force field (Merck Molecular Force Field) [148, 149].

### 5.3 General Docking Procedures

To evaluate the possible interaction of α‐humulene on GABA_A_ and 5‐HT_3A_ receptors, molecular docking simulations were performed using the human GABA_A_R structure (PDB 6HUP) [104] and the mouse 5‐HT_3A_R structure (PDB 6NP0) [150]. This choice is justified by the high structural homology between zebrafish and mammalian receptors: zebrafish GABA_A_R subunits share 60%–98% amino acid identity with their human orthologs [6], and the electrophysiological properties of these receptors are conserved among vertebrates [10]; likewise, the 5‐HT_3A_R gene is conserved across species, and mouse structures are routinely used as templates for modeling 5‐HT_3_ receptors in other organisms [3, 7]. Thus, these crystallographic structures provide a reliable basis for predicting ligand‐receptor interactions in the zebrafish model. The preparation of protein structures was performed using the AutoDockTools code [151], where missed residues were added to Kollman charges and polar hydrogen atoms [152, 153].

Molecular docking simulations were performed using AutoDockVina code [144], configured to run the Lamarckian Genetic Algorithm (LGA) and Exhaustiveness 64 [154]. Fifty independent simulations were performed using a simulation grid centered on the target in order to involve the entire protein structure with the axes: 125,281 (x), 139,534 (y), and 136,018 (z), size parameters 35Å (x), 35Å (y), and 35Å (z) with the GABA_A_R receiver and axes: ‐20.908595 (x), 10.200848 (y), and 21.639785 (z), size parameters 35Å (x), 35Å (y), and 35Å (z) with channel 5‐HT_3A_. To validate the docking simulations, a redocking procedure was performed using the co‐crystallized ligand PIO501 [153] for the GABA_A_ receptor, and the co‐crystallized ligand 2GM2001 for the 5‐HT_3A_ receptor.

The statistical parameter RMSD with values up to 2.0 Å [102] and affinity energy, with values close to −6.0 kcal/mol [155, 156], and the affinity energy was also used to assess the stability of the complexes formed. Using the values of the distances between the donor and acceptor atoms, it was evaluated the intensity of the hydrogen bonds (H‐Bond) classified as strong bonds when they present distances between 2.5–3.1 Å, average bonds between 3.1–3.55 Å and weak bonds when they present length greater than 3.55 Å [157].

### 5.4 Statistical Analysis of Behavioral Data

Analyses were carried out using the JASP 0.95.2.0 software. No data normalization nor transformation was applied, and outliers were not excluded; all data points were included in the analyses. Normality was assessed by the Shapiro–Wilk test (*p* > 0.05) and homogeneity of variances by Levene's test (*p* > 0.05). Data are expressed as mean ± standard error of the mean (SEM) for parametric data, or as Mdn with interquartile range (IQR) for nonparametric data. All groups had a number of six animals, therefore the total sample size for the statistical analysis of the locomotor activity in the open field test was 30 animals. For the statistical analysis of the anxiolytic‐like effect in the light/dark test was 30 animals. The sample size for each statistical analysis of the anxiolytic‐like effect under the action of each standard antagonist used in the light/dark test was 36 animals. The sex of the animals was not included as an independent factor, since previous studies do not indicate significant sex differences in the behavioral tests used and under the tested conditions [158, 159].

All statistical tests were one‐tailed with significance set at 5% (*p* < 0.05, *α* = 0.95, and *β* = 0.80). Data meeting normality and homogeneity of variance criteria were analyzed by one‐way ANOVA followed by Tukey's post–hoc test. Data with normal distribution but heterogeneous variance were subjected to one‐way ANOVA with Welch's correction, followed by Games–Howell post–hoc test. Non‐normally distributed data were analyzed by the Kruskal–Wallis test, followed by Dunn's post–hoc test. For overall group differences, omega squared (*ω* ^2^) [160] or eta squared (*η* ^2^) [161, 162] were reported as effect sizes. For specific pairwise comparisons, Hedges' g [163, 164, 165] (for parametric data), unbiased effect size “*e*” [166] (for normal distributed data with heterogeneous variance) or rank‐biserial correlation (rrb) [167, 168] (for nonparametric data) were reported. In the text, mean difference (Δ*M*), 95%CI, Mdn, IQR, mean rank (W), and Z‐score (z) are provided as appropriate.

## Author Contributions

The manuscript was written through equal contributions of all authors. All authors have given approval to the final version of the manuscript.

## Conflicts of Interest

The authors declare no conflicts of interest.

## Data Availability

Data will be available on request.
